# Prevention and management of behavioural and psychological symptoms in patients with dementia in acute care: a best practice implementation project

**DOI:** 10.1097/XEB.0000000000000329

**Published:** 2022-11-14

**Authors:** Rachid Akrour, Catherine Courret-Gilgen, Beatrice Perrenoud

**Affiliations:** 1 Acute Geriatric Care Unit; 2 Lausanne University Hospital; 3 BEST JBI Center of Excellence, Lausanne, Switzerland

**Keywords:** acute care, behavioural and psychological symptoms of dementia, dementia, nonpharmacological intervention, older adults

## Abstract

**Introduction:**

Behavioural and psychological symptoms of dementia are very common in acute care. Agitation and aggressive behaviours are the most common symptoms and are challenging to manage. Early detection and a nonpharmacological approach are recommended.

**Objectives:**

To implement evidence-based recommendations for the prevention and management of aggression/agitation in patients with dementia in an acute geriatric care unit.

**Methods:**

The project used the JBI Practical Application of Clinical Evidence System and Getting Research into Practice audit and feedback tool. A baseline audit was conducted with seven audit criteria based on evidence summaries. It was followed by the implementation of an action plan and a follow-up audit.

**Results:**

Results showed moderate improvements in compliance with best practice recommendations. The second audit indicated an improvement up to 46% with the identification of factors/triggers that precipitate aggression/agitation and completion of a risk assessment. The highest improvement was the training of the nursing team (79%). Compliance with the involvement of patients and their families in the care planning improved slightly (14%). The lowest improvement was for the development and implementation of individualized care plans (10%).

**Conclusion:**

The project implementation achieved some positive changes. A formalized process for preventing aggression/agitation is in place. The interprofessional collaboration, the support given to the nursing team and the basket of nonpharmacological interventions were strengthened. The electronic documentation and a limited collaboration of the nursing team were challenging. As further strategies were implemented, further audit would be required to assess achievement in change and/or demonstration of improved care provided for this vulnerable population.

**What is known about the topic?**

- Behavioural and psychological symptoms of dementia (BPSD) have a significant negative impact on the health of old patients, caregivers and healthcare professionals.
- Nonpharmacological approach is the recommended first line of treatment for the management of BPSD.
- Nonpharmacological interventions include adaptation of the physical care environment, development of the relationship with the patient and the caregivers, enhancing the skills of healthcare professionals and individualized care.

**What does this article add?**

- The practical conditions for using a reliable and valid assessment instrument of the risk of aggression/agitation (i.e. time-saving assessment and quick access to document it) should be carefully evaluated before its implementation.
- Nurses’ mental workload related to patient care with BPSD can be a barrier to their engagement in a quality improvement process.
- The increased involvement of other dementia professionals may be an avenue to relieve nurses of the mental burden and support them in complying with evidence-based practice recommendations.

## Introduction

The proportion of people with dementia in the world is more than 55 million and around 10 million are being added every year.^1^ In 2018, Switzerland's proportion of old adults with dementia was close to 148 000 and every year about 28 100 new cases are added.^2^ Behavioural and psychological symptoms of dementia (BPSD), such as anxiety, apathy, sleep disturbances, irritability, psychosis, wandering and agitation can occur in up to 90% in patients with dementia^3^ and are often a normal response to stress and uncertainty. The manifestation of BPSD varies according to the type of dementia. Manifestations, such as wandering, disinhibition, socially inappropriate behaviour and apathy are often associated with frontotemporal dementia. Depression is common in vascular dementia and hallucinations are likely associated with Lewy body dementia.^4^

Risk factors of BPSD are complex and multifactorial, and can be divided into biological, psychological and social or environmental factors. BPSD are also common in acute hospitals, with 75% of patients with dementia admitted with acute medical illness having BPSD.^5^ The hospital environment can be an exacerbating factor in the occurrence of BPSD. Various hyperstimulations and the stress generated by the care activities in the hospital can be factors precipitating aggression/agitation in patients with dementia.^6^ The occurrence of challenging behaviours, such as agitation/aggression varies among studies. Some authors mentioned that aggression/agitation are the most common symptom and are present in 76% of cases of BPSD.^7^ Other authors estimated that the aggressive behaviour occurs in 30–50% of patients with dementia.^8^

BPSD contribute significantly to the decrease in quality of life and increase severity of disability, malnutrition and nursing home admission among older adults.^7,9,10^ BPSD also have a significant impact on the physical and psychological well being of caregivers. The episodes of aggression and agitation are experienced as problematic and restrictive by family caregivers, thus amplifying the feeling of burden in their daily lives.^3^ Moreover, verbal or physical aggression management remains the most challenging task for healthcare teams. Aggression presents physical and psychological risks, which are often associated with negative healthcare professional outcomes, such as job dissatisfaction, staff turnover, stress and poor psychological health.^3,7,11^ During a hospitalization, BPSD are associated with increased use of healthcare, longer length of hospital stays and incur significant economic burden.^12^

According to JBI evidence-based recommendations,^13–16^ best practice for BPSD prevention in patients with dementia includes an early detection of the risk of aggression/agitation through a complete evaluation of the patient's situation using a standardized instrument, such as the Cohen-Mansfield Agitation Inventory (CMAI).^17^ A nonpharmacological approach is the first line treatment for management of BPSD whenever there are no imminent dangers or safety concerns.^15^ JBI recommendations include the functional analysis of BPSD, including determining and documenting the antecedents, behaviours and consequences of BPSD.^15,16^ A person-centred approach integrating the patient and their family or care team is essential to establish an individualized care plan focused on the patient's unmet needs and her/his environmental risk profile.^4,18,19^ A dementia-care training of healthcare professionals that is consistent with their role and responsibility is also recommended.^15,16,19^

Medication should only be used as part of a comprehensive treatment plan to address potential causes of BPSD and ensure the safety of patients and caregivers.^20^ Some studies mentioned the association of the use of psychotics with high risk of femur fracture, cardiovascular and cerebrovascular adverse events and mortality.^21–23^ A systematic review of reviews on nonpharmacological approaches for BPSD recommended sensory interventions, such as music therapy, light therapy and aroma therapy.^4^ Evidence on light therapy and aroma therapy is heterogeneous, whereas music therapy is the most convincingly effective intervention among sensory intervention in reducing behavioural symptoms.^4^ Family interventions for reducing BPSD were found to be appropriate for acute geriatric care.^4^ Family interventions may help to redesign the hospital environment.^4^ Organizational interventions, such as the development of care mapping or care plan, are based on a person-centred approach and can contribute to mitigate BPSD.^4,24^ A multidisciplinary approach combining medical, psychiatric and nursing interventions is recommended to detect, assess, treat and evaluate patients with dementia and can improve patient outcomes.^16^

The project presented in this report was undertaken in one of the five Swiss tertiary hospitals, which is both a primary care hospital and a referral hospital for a large part of French-speaking Switzerland, for specialized and ultra-specialized care. In 2019, the Lausanne University Hospital operated 1531 beds for around 51 205 hospitalized patients. In the same year, for the 22 980 people over 65 hospitalized, around 50% of them were 85 years and older. The university centre employed more than 5000 healthcare professionals and around 37% were nurses. The Acute Geriatric Care Unit (AGCU) of the Lausanne University Hospital consists of 28 beds, and around 600 patients per year are admitted in the unit. The criteria for admission to the AGCU to be 75 years or older, to have at least one geriatric syndrome and to require hospitalization in acute care. In 2015, the median length of AGCU stay was approximately 10 days. A brief retrospective analysis of nursing documentation of patients admitted in 2017 showed that 22 patients with obvious symptoms of aggression/agitation were hospitalized in the AGCU and their median length of stay was 27 days. This proportion increased to 31 patients in 2018 with a median length of stay of 31 days. A first brief review in the AGCU showed there was no systematic BPSD evaluation of admitted patients. This probably induced an underestimation of behavioural and psychological symptoms for dementia patients by healthcare professionals. Furthermore, there was no systematic use of nonpharmacological interventions for patients with BPSD. Due to the dramatic impact of BPSD for patients, relatives and healthcare professionals, it was important to incorporate systematic detection and prevention into the admission of patients.

To provide quality care to patients through the prevention and management of BPSD in order to avoid or at least limit the consequences of this syndrome on hospitalized patients with dementia, it is necessary to have an assessment of BPSD on admission, to develop and to implement an individualized care plan for each patient. The current project was conducted using JBI implementation methodology. The JBI implementation methodology involved the application of a clinical audit and feedback strategy, with the use of the JBI PACES (Practical Application of Clinical Evidence System) audit and feedback tool.^22^ The purpose of this project was to increase nursing team compliance with best practice recommendations on aggression/agitation management among patients with dementia and assess the impact of these practice changes in improving patient care. The stakeholders in this change of practice were the registered nurses and the Community Health and Care Assistants (a Swiss equivalent of assistant nurses) of the AGCU, as well as the management team composed of a head nurse and two clinical nurse specialist. Due to major changes in the medical team in that period, they could not be involved in the project but they supported it.

## Objective(s)

The project aimed to implement evidence-based recommendations for prevention and management of aggression/agitation in patients with dementia in an acute geriatric care unit.

The objectives were to: determine current compliance with best practice recommendations for prevention and management of aggression/agitation in patients with dementia, implement strategies to address identified gaps and improve compliance with evidence-based criteria for prevention and management of aggression/agitation in patients with dementia in the AGCU.

## Methods

The project used the JBI Practical Application of Clinical Evidence System (JBI PACES) and Getting Research into Practice (GRiP) audit and feedback tool. The JBI PACES and GRiP framework for promoting evidence-based healthcare involves preclinical and postclinical audits and feedback.

The project was registered as a quality improvement activity within the hospital, and therefore, did not require ethical approval. The Institutional Inquiry Assessment Board approved this project.

### Phase 1: stakeholder engagement, team establishment and baseline audit

In the first phase of this project, a core group of key stakeholders was formed to support the implementation of the project. The project team included:

(1) The Head Nurse of AGCU and a Clinical Nurse Specialist (CNS), were co-responsible for coordinating the project. They organized brainstorming sessions with the nursing team, planned and monitored progress, ensured deadlines and targets were met, and organized information sessions during the project.
(2) One Registered Nurse and one Community Health and Care Assistant, conducted audits and participated in the development of the project planning and targets, as well as strategies for GRiP analysis.
(3) The Nurse Manager (MN) of AGCU was the clinical governance and an executive sponsorship, ensuring the provision of necessary resources for healthcare training during the project implementation.
(4) A scientific development officer acted as methodological consultant to assist the project coordinators.

The objective of the baseline audit was to establish the importance and the nature of the gap between current practices and best practice recommendations in prevention and management of aggression/agitation in patients with dementia. In order to achieve the objective of the audit, seven audit criteria were generated by JBI on the JBI-PACES online tool for this project. They are based on best practice recommendations for nonpharmacological management of BPSD from JBI Evidence Summaries.^14–16^ The audit criteria were summarized in the existence of a process for the recognition, prediction, prevention and management of aggression/agitation, training of healthcare professionals in the detection and management of aggression/agitation in patients with dementia. They also included development and implementation of individualized care plans for prevention and management of aggression/agitation with the inclusion of patients and their families wherever possible. The seven audit criteria are presented in Table 1.

**Table 1:** Audit criteria

Audit criterion
(1) The organization has processes for recognizing, predicting, preventing and managing challenging behaviour.
(2) Healthcare staff receive education that allows them to recognize, predict, prevent and manage challenging behaviour.
(3) Healthcare staff, in consultation with patients, carers/families, identify factors/triggers that precipitate challenging behaviour.
(4) Patients undergo risk assessment by a health professional skilled in assessment and management.
(5) An individualized care plan is developed to prevent or manage the challenging behaviour.
(6) An individualized care plan is implemented to prevent or manage the challenging behaviour.
(7) Wherever possible, patients and their families are involved in the care planning.

The sample consisted of 28 patients and 34 healthcare professionals (registered nurses and Community Health and Care Assistants). All patients hospitalized in the unit on the day of the audit were included. The care environment, which is hyperstimulating, contributes to the risk of exacerbating the existing BPSD and/or of developing others. Due to hospitalization, every patient can be considered at risk of developing a BPSD. The detection of risk factors and antecedents is needed to anticipate aggression/agitation that are often a response to stress and uncertainty in a care environment.^4^ It was, therefore, important to know whether there was a systematic detection of the risk of BPSD for all patients since their entry to the unit, whether or not they have had an episode of BPSD. The project team developed two strategies to measure practices. The nurses and Community Health and Care Assistants collected the data at the patient's bedside by observation, and in the Electronic Health Records (EHR), using a grid (Table 2). The implementation of guideline and structured documents concerning the management of BPSD in the unit and the list of nursing team members present at the training sessions were used to assess criteria 1 and 2, respectively. Other criteria were assessed in the EHR, using a grid. To complete the audit of criteria 5, 6 and 7, a Registered Nurse and a Community Health and Care Assistant checked the rooms of the 28 patients present on the day of the audit. Using a grid, they observed whether elements were present in each patient's room. For example, if at least one nonpharmacological intervention (essential oil diffuser, music) could be observed; and if the patient's room or bedside was personalized (with pictures of familiar people, blanket, personal belongings, such as cushions or other important objects for the patient).

**Table 2:** Audit criteria, sample and approach to the measurement of compliance with best practice

Audit criterion	Sample	Method used to measure percentage compliance with best practice
(1) The organization has processes for recognizing, predicting, preventing and managing challenging behaviour.	1 Acute Geriatric Care Unit (AGCU)	Guidelines and structured documents used in the units
(2) Healthcare staff receive education that allows them to recognize, predict, prevent and manage challenging behaviour.	34 AGCU nurses and CHCA, Community Health and Care Assistant.	Attendance sheet at the training sessions
(3) Healthcare staff, in consultation with patients, carers/families, identify factors/triggers that precipitate challenging behaviour.	28 patients	At least one factor/trigger is cited and/or documented: At the patient's bedside: **YES/NO** In the EHR: **YES/NO**
(4) Patients undergo risk assessment by a health professional skilled in assessment and management.	28 patients	The Cohen Mansfield Agitation Inventory evaluation has been completed within 48 h of admission: At the patient's bedside: **YES/NO** In the EHR: **YES/NO**
(5) An individualized care plan is developed to prevent or manage the challenging behaviour.	28 patients	At least one nonpharmacological intervention or one individualized action planned:At the patient's bedside: **YES/NO** In the EHR: **YES/NO**
(6) An individualized care plan is implemented to prevent or manage the challenging behaviour.	28 patients	At least one nonpharmacological intervention or one individualized action provided:At the patient's bedside: **YES/NO** In the EHR: **YES/NO**
(7) Where possible, patients and their families are involved in the care planning.	28 patients	At least one nonpharmacological intervention resulting from discussion with the patient/family is cited or documented:At the patient's bedside: **YES/NO** In the EHR: **YES/NO**

Nurses and the Community Health and Care Assistants conducted the baseline audit on January 7 2019. The data were entered into the JBI PACES program and a compliance report was generated.

### Phase 2: design and implementation of strategies to improve practice (Getting Research into Practice)

The project was presented to all members of the AGCU healthcare team (nurses, Community Health and Care Assistants, physicians, physiotherapists) in September 2018. During a meeting in March 2019, the baseline audit results were presented to the nursing team, followed by discussion. During that session, the GRiP module of JBI PACES was used to identify barriers to compliance with best practice recommendations and resources required to develop and implement strategies to improve practice. Strategies were developed with the nursing team present at the March session to improve compliance with best practice and reported in Table 3.

**Table 3:** Getting Research into Practice matrix

Barrier	Strategy	Resources	Outcomes
Lack of knowledge on recognizing, preventing and managing BPSD	Training of the healthcare professionals in detection, prevention and nonpharmacological management of BPSD	Geriatric psychologist	Increased knowledge on detecting, predicting and managing agitation/aggression. 80% of healthcare staff trained.(criterion 2)
Lack of systematic assessment of BPSD at patient's admission	Integrating systematic Cohen Mansfield agitation inventory (CMAI) assessment at patient's admission	CMAI available in the Electronic Healthcare Record (EHR) and coaching for the use of CMAI insured by CNS and RN	Increased identification of patient at risk of agitation/aggression (criteria 1, 3, 4)
Lack of continuity in the implementation of nonpharmacological interventions	Making nonpharmacological measures available to the healthcare staff and informing them.Engaging families and nursing team in implementing tailored interventions for the patients	Aromatherapy, music therapy, hypo-stimulating space (lounge), customization of rooms with patient objects or photos	Increased variety of nonpharmacological interventions available and use of interventions (criteria 1, 5, 6, 7)
Dissatisfaction with the work process to prevent and manage BPSD:- gaps in development of care plans and their follow-up.- gaps in communications between the daily nurses and CHCA	Establishing a common care plan (nurse/CHCA) for patients at risk of BPSD.Discussion about challenging situations between CNS, HN and nursing staff to propose the best care plan.Involving experts like CNS, psychologist or psychogeriatric in these situations	Morning nursing handover.Psychologist, Psychogeriatric	Improved follow-up audit results for (criteria 1, 5, 6, 7)
Lack of nursing documentation of risk factors for BPSD and nonpharmacological interventions in EHR	Implementing a protocol for a systematic documentation of risk assessment and nonpharmacological management	Local team work	Increased documentation of behaviour management (criterion 1)
Trivialization of aggressive behaviour	Systemic reporting of aggression events of patients with BPSD over 24 h using the institutional tool for reporting adverse events.Discussion of aggression/agitation situations between medical and nursing staff and establishment of care strategies.	Institutional collection of critical and undesirable events system.Geriatric psychologist.NM	Decreased commoditization of aggressive behaviour (criterion 1)

BPSD, behavioural and psychological symptoms of dementia; CNS, Clinical Nurse Specialist; HN, head nurse; NM, nursing manager.

The nursing team identified gaps in knowledge and skills of managing aggression/agitation in the AGCU. They also reported their needs for additional training. Moreover, the nursing team reported the absence of a structured care process for patients at risk at the time of admission as well as during implementation and monitoring of the nonpharmacological interventions.

In March 2019, an action plan was developed with the stakeholders and implemented the same month. The action plan aimed to ensure the training of at least 80% of the nursing team, and implement the CMAI^17^ as a detection tool. The choice of the tool was based on the evidence-based recommendations^13,14^ and its availability in the institutional EHR. The action plan also promotes the use of nonpharmacological interventions and improves the documentation of BPSD management by the nursing team. The follow-up audit was performed 4 months after implementation, to allow enough time for the planning and delivery of the four training sessions.

### Phase 3: follow-up audit postimplementation of change strategy

A follow-up audit was carried out 4 months after the implementation phase, using the same seven criteria, grid and questionnaire used in phase 1. There were no variations to the topic, criteria, sample size or location during the follow-up cycle. The objective of the follow-up audit was to assess whether any improvement in compliance with the best practice had been achieved and to identify any areas requiring further improvement.

The follow-up data were entered into the JBI-PACES program and a compliance report was generated comparing them to the baseline audit results. Changes in compliance were measured using descriptive statistics embedded in JBI-PACES in the form of percentage changes from baseline.

## Results

### Phase 1: baseline audit

**Figure 1:**
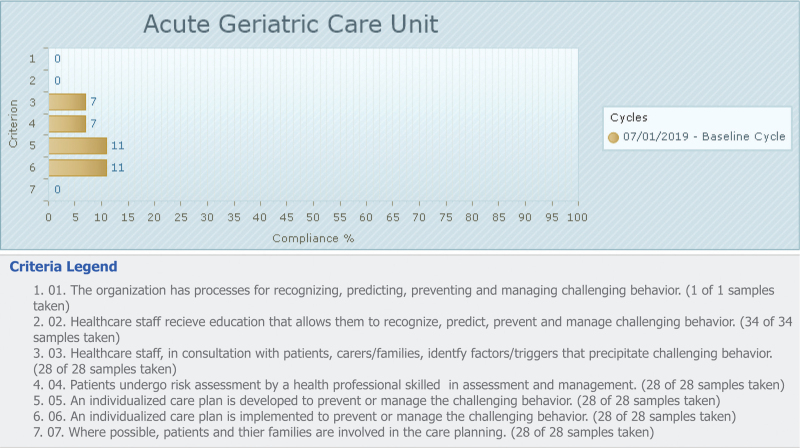
Baseline compliance with best practice for audit criteria (percentage).

The aggregated compliance percentages for each criterion from the baseline audit are reported in Fig. 1.

Performance emerged as very poor in the baseline audit for all criteria. The best baseline performance was found for criteria 5 and 6, which each displayed 11% compliance for developing and implementing individualized care plan, respectively.

The lowest rate emerged for criteria 1, 2 and 7 with 0% compliance regarding processes for recognizing, predicting, preventing and managing aggression/agitation (criterion 1), nursing staff education in preventing, predicting and managing aggression/agitation as well as with the involvement of patients and their families in care planning.

A 7% compliance was observed for criterion 3 (healthcare staff in consultation with patients, carers/families, identified factors/triggers that precipitate aggression/agitation), and criterion 4 (patients undergo risk assessment by a health professional skilled in assessment and management).

### Phase 2: strategies for Getting Research into Practice

The barriers identified by the nursing team during the March session made it possible to set up an action plan with the resources already available in the unit (Table 3).

For each barrier, strategies have been implemented:

(1) A 45 min training course was developed and delivered by a geriatric psychologist. The content of the course included information on the prediction, prevention and management of agitation/aggression in patients admitted to the unit. It also included clinical vignettes for participants to think of and find possible solutions to aggression/agitation. The training course was repeated on four occasions to include at least 80% of the unit's healthcare staff. The Head Nurse planned the training sessions and monitored the attendance to the sessions.
(2) The CMAI was implemented as a standardized tool to identify patients at risk of agitation/aggression and identify factors/triggers that precipitate BPSD.
(3) The existing nonpharmacological interventions, such as spatial orientation, securing doors and music therapy were strengthened by introducing aromatherapy with essential oil diffusers. The patient lounge was also refurbished to provide a calm and hypostimulating environment for patients and their families. Wherever possible, families and caregivers were involved in the personalization of the patient's rooms with pictures and other personal items to create a reassuring environment.
(4) The documentation of aggression/agitation assessment, risk factor identification with the patient and his/her caregiver and nonpharmacological interventions were integrated into routine nursing care. A working group of nurses and Community Health and Care Assistants was in charge of developing systematic documentation in the EHR. The topic of aggression/agitation evaluation and nonpharmacological interventions became a priority for the AGCU.
(5) Situations of patients with challenging behaviour were discussed during the morning nursing handover. The Head Nurse ensured the measures documented in the care plan were implemented and encouraged nursing staff to ask for expert referral, such as psychologist, psychogeriatric CNS or psychogeriatric when judged necessary.
(6) The nursing staff was encouraged by the MN, Head Nurse and CNS to report aggressive events related to the BPSD in the institutional system for reporting adverse events in order not trivialize these events by the healthcare team and generate a global and institutional reflection on the improvement and management of the aggression/agitation.
(7) Interdisciplinary meetings were set up (with nurses, physicians and geriatric psychologist), and where situations of patients with aggression/agitation were discussed and analysed to find common strategies to manage these situations.

### Phase 3: follow-up audit(s)

**Figure 2:**
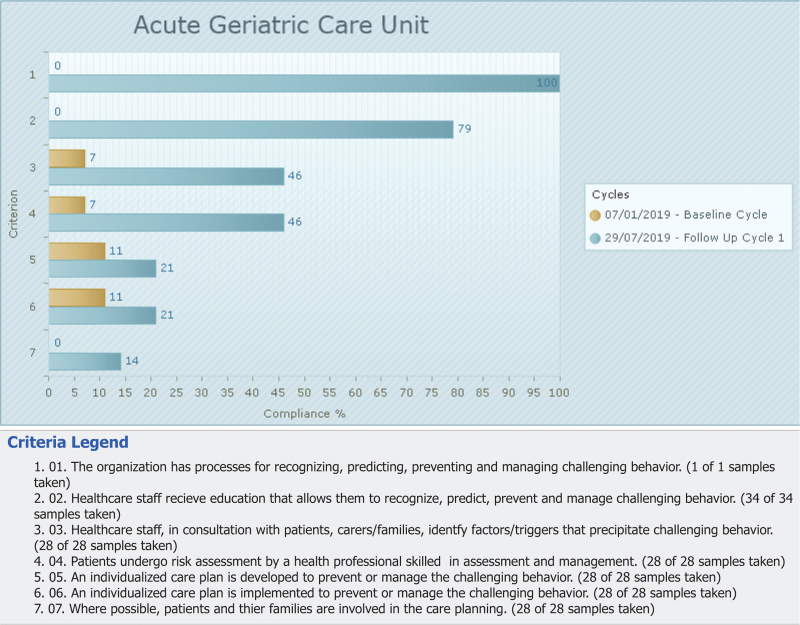
Compliance with best practice for audit criteria in follow-up audit compared with baseline audit (percentage).

The percentage of compliance for the audit criteria for the follow-up audit and the baseline audit are displayed in Fig. 2. In general, the results of the follow-up audit, compared with those in the baseline audit, showed an overall improvement in compliance of implementing best practice in prevention and management of aggression/agitation in patients with dementia. The most notable improvement was for criterion 1 indicating that organization has processes for the recognition, prediction, prevention and management of aggression/agitation (100%), and for criterion 2 measuring that healthcare staff received education and training in preventing, predicting and managing aggression/agitation, which achieved 79% compliance.

The following criteria improved from baseline (7%) to follow-up audit (46%): healthcare staff, in consultation with patients, carers/families, identified factors/triggers that precipitate aggression/agitation (criterion 3) and patients underwent risk assessment by a health professional skilled in assessment and management of aggression/agitation (criterion 4).

Criteria 5 and 6 related to the elaboration and implementation of an individualized care plan showed a relative improvement in compliance best practice from 11 to 21%. The lowest compliance best practice in the follow-up audit was observed in the criterion measuring the involvement of patients and their families in the care planning (criterion 7), although this criterion improved from 0 to 14%.

## Discussion

The implementation project was conducted in the acute geriatric unit of a Swiss tertiary hospital to promote evidence-based aggression/agitation prevention and management in patients with dementia among the nursing staff. The implementation project has resulted in moderate overall improvements in compliance with best practice recommendations. The project set up a formalized process for patients at risk of aggression/agitation (100%, criterion 1), strengthening nursing staff's focus to BPSD^6,16^ and assisting nursing staff in improving the prevention of BPDS. Aggression episodes were systematically reported as adverse events, which demonstrates the importance of such events by nursing staff.^15,16^

The literature identified the need for specialized training for nurses to provide the care required by BPSD symptoms.^11^ Before the project, very few nursing staff members were trained in aggression/agitation prevention and management in patients with dementia. The four-session training permitted the training of 79% of the nursing staff (criterion 2). The timing of these training courses during day shift is likely to have contributed to the targeted participation rate. The training allowed an improvement in nursing staff's knowledge and skills. The use of these skills for the benefit of patients was shown by an increase from 7 to 46% in the identification of factors that precipitate agitation/aggression and the risk assessment (criteria 3 and 4) in the follow-up audit. Identification of precipitating factors and risk assessment are essential prerequisites for the implementation of an individualized care plan in order to avoid or minimize episodes of agitation/aggression during hospitalization.^4,13–16^ Specific training on patient-centred care could reinforce the importance of assessment to prevent agitation/aggression in patients with dementia.^16^

The project itself also contributed to improve interprofessional collaboration. The geriatric psychologist was more involved in the aggression/agitation situations contributing to maintain the quality of life of patients.^7,9,20^ Multidisciplinary healthcare team meetings with physicians, psychologists and nurses were organized for most demanding situations of patients with aggression/agitation to discuss the best care strategies.^15^

The development. and implementation of individualized care plan for patients (criteria 5 and 6), including the use of nonpharmacological interventions to prevent or manage aggression/agitation, slightly increased from 11 to 21%. This could indicate that nursing staff did not consider it necessary to deploy a care plan for all patients assessed but only for those deemed to be at risk of becoming agitated/aggressive. With this implementation project, we did not identify how many patients exhibited BPSD or aggression/agitation behaviour in the samples of the baseline and follow-up audits. This may have influenced the audit results in terms of care plans implemented. In future audits, it would be useful to identify the characteristics of the patients in order to give more precise feedback to the nursing team on their practices. In any case, a small increase in individualized care plan is still clinically important, as patients, caregivers and professionals can be affected by aggression/agitation.^11^ For the nurses, such symptoms are time-consuming, needing to constantly monitor the patient, having an impact on the care of other patients.^11^ Individualized coaching, mentoring, supervision and team meeting, and the integration of this theme into existing training should be considered to strengthen prevention practices of BPSD for all patients.^18^

The compliance for criterion 7 relating to involvement of patients and their families in the care plan showed a slight increase from 0 to 14% in the follow-up audit. These results were disappointing as they do not seem to reflect nursing practice in which nursing exchanges with patients and their families are common and the information collected is usually integrated into the patient's daily care. It can be assumed that before the implementation of the project, the nursing staff members underestimated all information collected from exchanges with patients and their families concerning aggression/agitation, which can be explained by poor documentation of these two conditions in patient care plans. However, even after four sessions of training provided in prevention and management of aggression/agitation, the documentation of nursing interventions remained suboptimal. This could be because of the persistent underestimation of the importance to document the information collected from the patients and their families by the nursing staff for continuity of care.

Three other main reasons could explain the results regarding the documentation of an individualized care plan for the patients and their families (criteria 5–7). The first limitation is related to the use of the CMAI in its electronic form, which is too long and poorly structured compared with the paper version that fits on one page. The electronic format discouraged the nursing team to complete it systematically. The coaching provided by the CNS and the Registered Nurse to use the CMAI proved not to be sufficient to overcome this barrier. The second issue was the inability for nurses to document the nonpharmacological interventions in agitation/aggression prevention and management directly into the EHR. The system was designed in a way that does not allow for additional records. The literature showed that the design of EHR has an important influence on healthcare professionals’ documentation and compliance with best practice recommendations.^23^ The working group in charge of creating the systematic documentation of aggression/agitation care interventions had not developed a documentation procedure for the EHR before and after the follow-up audit, because of a lack of availability. These limits were discussed with the Information Technology team. Due to the limited staff and a large number of requests, the Information Technology was not able to modify the nursing documentation forms prior to the follow-up audit. As a result, the documentation certainly did not reflect all nursing practices and the EHR was a limitation of the project.

The third barrier could be related to the postponement of nursing staff training. The training was indeed the last strategy of the action plan to be deployed because of the unavailability of the trainer. The four training sessions took place, when the implementation of aggression/agitation management had already begun in the unit. This delay may have influenced the results regarding the implementation of an individualized care plan for patients with dementia. It would have been more appropriate to provide the nursing team training before implementing the change of management of aggression/agitation in clinical practice.

Results of the follow-up audit were analysed and discussed with the nursing team members, who identified two new barriers to compliance with evidence-based practice. The first barrier was that the nursing team expressed feeling uncomfortable caring for patients with agitation/aggressive behavior. The nursing staff felt that the mental workload was a burden. Professionals noted that many patients with BPSD on the AGCU should have been in a psychogeriatric unit rather than in a geriatric one. These patients present complex care problems for which there are not always solutions.^19^ The care of these patients is demanding, and often requires one-to-one care. Nurses often suffer from stress and frustration providing care for patients with dementia. They may become emotionally numb or feel anger and guilt.^11^ More ongoing support to staff nurses could reduce mental workload.^11^ The second barrier was the lack of communication between the nursing staff members in developing an individualized care plan for a patient and ensuring continuity of care. In spite of benefiting from a dedicated room and time to formalize nursing communication about the unmet needs and common goals of care for a patient, the nursing team could only come to an agreement when the head nurse was present. This confirms that nurse managers are considered as primary gatekeepers for evidence-based practice. They can involve staff and provide a supportive environment in the implementation of evidence-based practices, with responsive management and effective leadership.

A new action plan was developed. Some strategies to reassure and support the nursing staff and to improve the care processes and documentation were chosen:

(1) Reinforce listening during the healthcare meetings in order to defuse the mental workload in the nursing staff when there is a patient with agitation/aggressive behaviour.
(2) Support close collaboration with the psychogeriatric CNS in supporting the healthcare team in preventing and managing patients with agitation/aggressive behaviour.
(3) Organize meetings with the nursing staff to address the problem of lack of collaboration around care plans and nursing activities.
(4) Reinforce follow-up of patients with agitation/aggressive behaviour from medical staff and the geriatric psychologist.
(5) Use the psychiatric liaison nurse to distract the patient with agitation/aggressive behaviour during painful or long care (e.g. dressing).
(6) Reinforce a low environmental stimulation.
(7) Adapt pleasant occupations to the patient from the moment he or she is admitted.
(8) Simplify the documentation and create a paper form that makes all the interventions of the nursing staff easier to complete and visible.
(9) Be cautious to balance patient with dementia quota in the unit and to reinforce staffing during the night, when the nurse/patient ratio is lower.

To ensure the sustainability of best practice recommendations, the training course is planned twice a year for new staff, and an on-the-job mentorship is provided during the first month in the job. A public presentation of the project resulted in getting funding to develop more nonpharmacological approaches. This funding also provided recognition and support to nursing staff members in their mission of providing care to patients with dementia.

If the second action plan has been deployed, a new follow-up audit has not been carried out in order to formally demonstrate the performance achieved in preventing and managing agitation/aggression. A big challenge for stakeholders was to conduct this project in the middle of several other projects deployed in the institution and impacting the ACGU. Stakeholders were highly committed to the sustainability of the strategies implemented to improve aggression/agitation prevention and management, the nursing staff support, and the increase of nonpharmacological interventions. Stakeholders felt that they have no time to conduct another audit. The time pressure they felt may mean that they do not perceive practice monitoring to be a priority. This is still a challenge in our hospital as the monitoring of healthcare performance indicators is not a standard practice. If lessons can be learned from this implementation experience, the project was conducted with a small sample of patients, in a specific context. Results are not generalizable.

## Conclusion

Prevention and management of aggression/agitation in patients with dementia is crucial in acute care settings. They not only ensure the quality and safety of care for an already vulnerable population but also reduce the iatrogenic risks inherent to their hospitalization. The purpose of this project was to increase nursing team compliance with best practice recommendations related to prevention and management of aggression/agitation in patients with dementia in an acute geriatric care unit. The project showed relative improvements in nursing team compliance with best practice recommendations, requiring the implementation of a new action plan. The project faced many challenges, such as quick access and adequate format for the documentation of the BPSD screening tool and nonpharmacological interventions in the EHR. However, improvements to the documentation were not possible during the project. The perceived mental workload and the lack of nursing collaboration limited development of individualized care plans for patients with dementia common to all nurses. The project also faced a common challenge in care, that is conducting several projects at the same time, which possibly generated some fatigue within the nursing team. Nevertheless, practice performance should still be re-audited. The project has shown benefits in strengthening interprofessional collaboration and the support given to nursing staff members around the management of aggression/agitation, contributing to improved processes of care.

## Acknowledgements

The authors would like to thank all healthcare staff of the AGCU. The authors would like to acknowledge the team members involved in this project: Rime Tabia, Community Health and Care Assistant, Lausanne University Hospital and Christophe Oliveira Da Silva, RN, Lausanne University Hospital. The authors would also like to thank Professor Christophe Büla and Doctor Marc Humbert, Geriatricians, Lausanne University Hospital; Anne Véronique Dürst, Psychologist, Lausanne University Hospital, Carla Gomes da Rocha, School of Health Sciences, HES-SO Valais-Wallis, Professor Anne-Sylvie Ramelet, University of Lausanne, for her valuable proofreading, and to the BEST training team and all training colleagues.

Funding: the authors received no financial support for the implementation project and the publication of this article.

### Conflicts of interest

There are no conflicts of interest.
